# Supplementary material for: The impact of leishmaniasis on mental health and psychosocial well-being: A systematic review
Source: PLoS One. 2019 Oct 17;14(10):e0223313. doi: 10.1371/journal.pone.0223313 (PMC6797112; doi:10.1371/journal.pone.0223313)
Supplement: S1 PRISMA Flow Diagram — Out of 362 Abstracts that conformed to the inclusion criteria, 45 full articles were assessed for eligibility. 14 final articles were selected for analysis. (DOC) [file pone.0223313.s002.doc]

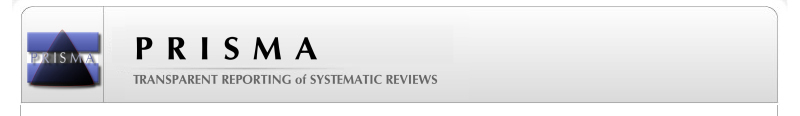
**PRISMA 2009 Flow Diagram**

**Screening**

**Included**

**Eligibility**

**Identification**

Records identified through database searching
(n = 17,890 )

Additional records identified through other sources
(n =1 )

Records after duplicates removed
(n = 12517 )

Titles screened
(n =12517 )

Full-text articles assessed for eligibility
(n = 45 )

Full-text articles excluded, with reasons
(n =31 )

Studies included in qualitative synthesis
(n =14 )

Studies included in quantitative synthesis (meta-analysis)
(n = 0 )

Titles excluded
(n =12155 )

Abstracts screened
(n =362 )

Abstracts excluded
(n =307 )
