# Supplementary material for: The impact of leishmaniasis on mental health and psychosocial well-being: A systematic review
Source: PLoS One. 2019 Oct 17;14(10):e0223313. doi: 10.1371/journal.pone.0223313 (PMC6797112; doi:10.1371/journal.pone.0223313)
Supplement: S1 Search Strategies — (DOCX) [file pone.0223313.s003.docx]

**Search Strategies**

**EMBASE, MEDLINE, PsycInfo**

1. (leishmani$ or kala-azar or kalaazar).mp.
2. cogniti*.mp.
3. Cognitive Dysfunction/
4. Disability Evaluation/ or Disabled Persons/ or disabilit*.mp.
5. physical impairment*.mp.
6. ?morbid*.mp. or exp MORBIDITY/ or exp comorbidity/
7. coinfect*.mp or exp co-infection/
8. exp mental disorders/ or mental disorders.mp.
9. exp mood disorders/ or mood.mp.
10. exp fear/ or fear.mp.
11. Feeling*.mp.
12. exp compulsive behavior/ or compulsive behavior.mp. or exp behavior, addictive/ or addict*.mp.
13. (psych* not psychodidae).mp
14. bipolar disorder/ or bipolar.mp.
15. exp cyclothymic disorder/ or cyclothymic.mp.
16. exp affective disorders/ or affective disorders.mp
17. (depressive adj2 (symptom* or disorder*)).mp.
18. exp depressive disorder/ or depression/ or depression.mp.
19. postpartum depression.mp. or exp Depression, Postpartum/
20. exp Stress, Psychological/
21. exp panic disorder/ or stress disorders.mp. or panic.mp. or stress.mp.
22. exp anxiety disorders/
23. (suicid* or mood or ((affect or affective) adj2 disorder*) or mani* or depressi* or neuros?s or neurotic* or adjustment disorder* or anxiet* or anxious or distress).mp.
24. exp Ajudstment disorders/ or adjustment disorder*.mp.
25. exp Neurotic disorders/ or neurotic.mp.
26. exp personality disorders/ or Personality disorder*.mp.
27. exp schizophrenia/ or schizoph$.mp.
28. exp Dysthymic Disorder/ or dysthym*.mp
29. self-esteem.mp. or exp Self Concept/
30. exp "quality of life"/ or quality of life.mp.
31. (disfigurement* or scar* or deform*).mp.
32. exp Social Discrimination/ or exp Prejudice/ or exp social exclusion/
33. (prejudice or exclusion or exclud*).mp.
34. exp SOCIAL STIGMA/ or stigma.mp.#
35. marginalization.mp.
36. discrimination.mp
37. ((Health Knowledge, Attitudes, Practice/ or health.mp.) adj2 knowledge.mp.) or attitude*.mp.
38. misconception*.mp.
39. ($understand* or misunderstand*).mp.

1. (rejection or shyness or social adjustment or social isolation).mp.
2. exp "Rejection (Psychology)"/
3. exp Social Adjustment/
4. exp Social Isolation/
5. exp Stereotyping/ or exp Self Concept/
6. (stereotyp* or self-concept or self-esteem).mp.
7. social norm*.mp.
8. (body adj2 satisfaction).mp.
9. poverty.mp. or poverty/
10. socioeconomic status.mp. or exp social status/ or SES.mp
11. education.mp. or exp education/
12. cultural.mp.
13. victim*.mp. or exp victim/
14. fatigue.mp. or exp fatigue/
15. (burden adj2 financial).mp
16. (burden adj2 family).mp
17. shame/ or ?shame*.mp or guilt/ or guilt*.mp
18. humiliat*.mp
19. avoid*
20. conceal*
21. or/2-59
22. 1 AND 60

**LILACS**

(tw:("kala-azar" or "kalaazar" or leish*)) AND (tw:(psic* or psych* or stigma or estigma or cicatri* or scar* or educa* or mood or humor or stress or ansiedade or anxiety or depress* or cognit* or panic* or adjustment disorder or discrimin* or attitude* or atitude* or self-esteem or auto-estima or reject or rejeição or victim* or "quality of life" or "qualidade de vida" or "calidad de vida" or comorbid* or morbid* or coinfec* or poverty or pobreza or "socioeconomic status" or "SES" or fatigue or fadiga))

**Global Health, IndMED, African Index Medicus, ArabPsyNet**

“leishmaniasis” or “leishmaniose” or “kala-azar” or “kalaazar”
