# Supplementary material for: The impact of leishmaniasis on mental health and psychosocial well-being: A systematic review
Source: PLoS One. 2019 Oct 17;14(10):e0223313. doi: 10.1371/journal.pone.0223313 (PMC6797112; doi:10.1371/journal.pone.0223313)
Supplement: S1 Table — (DOCX) [file pone.0223313.s004.docx]

| **Study** | **Selection** | | | | **Comparability** | **Outcome/Exposure** | | |  | **Total # stars** | **Quality rating** |
| --- | --- | --- | --- | --- | --- | --- | --- | --- | --- | --- | --- |
| **Cross-sectional** | Is sample representative? | Sample size | Non-respondents | Ascertain-ment of exposure | Control of confounding factos | Assessment of outcome | Statistical test | - |  |  |  |
| Alemayehu et al 2017 | * | * |  | ** | ** | ** | * | - |  | 9 | Good |
| Chahed et al 2016 | * |  |  |  |  | ** |  | - |  | 3 | Poor |
| de Castro Toledo et al 2013 |  |  |  | ** | * | ** |  | - |  | 5 | Fair |
| Govil et al 2018 | * |  | * | * | ** | ** | * | - |  | 8 | Good |
| Handjani et al 2013 | * |  |  |  |  | ** |  | - |  | 3 | Poor |
| Honório et al 2016 | * |  | * |  | ** | ** |  | - |  | 6 | Fair |
| Layegh et al 2017 | * |  |  |  |  | ** |  | - |  | 3 | Poor |
| Simsek et al 2008 | * | * |  |  | ** | ** | * | - |  | 7 | Fair |
| Vares et al 2013 | * |  |  |  |  | ** |  | - |  | 3 | Poor |
| **Case-control** | Is case definition representative adequate? | Are cases? | Control selection | Control definition | Comparability of cases and controls | Ascertain-ment of exposure | Same method of ascertainment for cases and controls | Non-response rate |  |  |  |
| Pal et al 2017 | * | * | * | * | ** |  | * | * n/a |  | 8 | Good |
| Turan et al 2015 | * | * | * | * | ** |  | * | * n/a |  | 8 | Good |
| Yanik et al 2004 | * | * | * | * | ** |  | * | * n/a |  | 8 | Good |
| **Cohort** | Is exposed cohort representative? | Selection of non-exposed cohort | Ascertain-ment of exposure | 4 outcome present at start? | Comparability of cohorts | Assessment of outcome | Follow-up long enough? | Adequacy of follow-up |  |  |  |
| Alemayehu et al 2018 | * | * | * | * | ** | * | * | * |  | 9 | Good |

**S1 Table- Newcastle Ottawa Scale**

**AHQR quality threshold conversion:**

**Good quality:** 3 or 4 stars in selection domain AND 1 or 2 stars in comparability domain AND 2 or 3 stars in outcome/exposure domain

**Fair quality:** 2 stars in selection domain AND 1 or 2 stars in comparability domain AND 2 or 3 stars in outcome/exposure domain

**Poor quality:** 0 or 1 star in selection domain OR 0 stars in comparability domain OR 0 or 1 stars in outcome/exposure domain

n/a- not applicable
