# Supplementary material for: The impact of leishmaniasis on mental health and psychosocial well-being: A systematic review
Source: PLoS One. 2019 Oct 17;14(10):e0223313. doi: 10.1371/journal.pone.0223313 (PMC6797112; doi:10.1371/journal.pone.0223313)
Supplement: S2 Table — (DOCX) [file pone.0223313.s005.docx]

**S2 Table -ROBIS Phase 1 Assessing Relevance**

| **Phase 1** | **Target Question** |
| --- | --- |
| Aetiology reviews |  |
| Patients/Populations | Persons (patients or their relatives) who have experienced a skin condition linked to cutaneous leishmaniasis |
| Exposures | LCL- related stigma |
| Outcomes | Psychosocial burden |

Phase 1 of assessment of methodological quality using ROBIS tool for aetiology reviews.
