# Supplementary material for: The impact of leishmaniasis on mental health and psychosocial well-being: A systematic review
Source: PLoS One. 2019 Oct 17;14(10):e0223313. doi: 10.1371/journal.pone.0223313 (PMC6797112; doi:10.1371/journal.pone.0223313)
Supplement: S3 Table — (DOCX) [file pone.0223313.s006.docx]

### **S3 Table- ROBIS Phase 2 Identifying concerns about bias in the review process**

| **Domain 1** | **Domain 2** | **Domain 3** | **Domain 4** |
| --- | --- | --- | --- |
| **1.1**Did the review adhere to pre-defined objectives and eligibility criteria? **PN**  There was no published protocol. Without a published protocol this is a difficult assumption to make as the authors could have written anything in the paper retrospectively. | **2.1** Did the search include an appropriate range of databases/electronic **PN**  A hand search on the reference list of the selected articles and searches for any publication by the first, second, third and last authors of the selected articles that met the same inclusion criteria but that were not initially detected by the initial search strategy. | **3.1** Were efforts made to minimise error in data collection? **PN**  It is not clear how data was obtained. | **4.1** Did the synthesis include all studies that it should? **PN**  Narrative synthesis cannot be assumed to be appropriate because without a published protocol we cannot tell if all relevant studies were captured. |
| - 1. Were the eligibility criteria   appropriate for the review question? **PY**  Research question was: Do persons experiencing a LCL skin lesion suffer from any psychosocial burden? Eligibility criteria was: Population- persons (or their relatives) experiencing a skin condition linked to LCL. Range of concepts- LCL related stigma and its psychological and social consequences in different LCL settings. Context: All countries. All types of publication were eligible. However they do exclude articles for which no full text was available and also exclude books. This could be potentially limiting the amount of information found. | **2.2** Were methods additional to database searching used to identify relevant reports? **Y**  A hand search on the reference list of the selected articles and searches for any publication by the first, second, third and last authors of the selected articles that met the same inclusion criteria but that were not initially detected by the initial search strategy. | **3.2** Were sufficient study characteristics available for both review authors and readers to be able to interpret the results? **Y**  Yes. Enough study characteristics were extracted for both authors and readers to interpret results. | **4.2** Were all predefined analyses followed or departures explained? **N**  No published protocol. |
| - 1. Were research criteria unambiguous?  **PY**   Research criteria was not ambiguous. It was clear what the authors were going to search for. There are low concerns regarding clarity. Very detailed inclusion of study designs and type of publications. No specific details about how patients/population were to be diagnosed with LCL or how they would know that a skin condition was linked to LCL. | **2.3** Were the terms and structure of the search strategy likely to retrieve as many eligible studies as possible?  **PY**  Eligible studies had to follow the following criteria: Population- persons (or their relatives) experiencing a skin condition linked to LCL. Range of concepts- LCL related stigma and its psychological and social consequences in different LCL settings. Context: All countries. All types of publication were eligible. For population, the search strategy was very comprehensive in that it referred to all types of cutaneous leishmaniasis as well as MCL (but leaving out VL). It was a good idea to be as sensitive as possible by using relevant synonyms, MESH terms. However, date and language restrictions were applied which could potentially cause selection bias. | **3.3** Were all relevant study results collected for use in the synthesis? **PY**  A lot of relevant data was extracted from the primary studies and described in both text and tables (Tables 1, 2 and 3). | **4.3** Was the synthesis appropriate given the nature and similarity in the research questions, study designs and outcomes across included studies? **Y**  Because of the heterogeneity of the studies, narrative synthesis only was appropriate. |
| 1.4Were all restrictions in eligibility criteria based on study characteristics appropriate? **Y**  Studies to be included were those on humans only. Studies with indirect information about psychological or psychosocial outcomes were to be excluded as were those on HIV and mental illness or other stigmatizing disease without a link to CL. This seems appropriate. Again, restricting articles without full text and books could be potentially limiting. Articles without entire text can usually be retrieved. | 2.4 Were restrictions based on date, publication format, or language appropriate? **PN**  The date restriction was from 1st Jan 1920 to 31st Aug 2017. It was almost 100 years but it could have missed previous epidemics. Included languages missed Portuguese where cutaneous leishmaniasis is also highly endemic. | 3.4 Was risk of bias or methodological quality formally assessed using appropriate criteria? **N**  No quality appraisal or ROB tool was used at all in this review. | 4.4 4 Was between-studies variation  (heterogeneity) minimal or addressed in the synthesis?  **PY**  The article references between-studies variation. |
| 1.5 Were any restrictions in eligibility criteria based on sources of information appropriate? **PY**  Most restrictions were appropriate, such as lab-based studies, articles targeting veterinary studies and articles about the vectors and not leishmaniasis itself. Restricting based on availability of full text as well as excluding books could induce selection bias, as these may be published in lesser known journals that are harder to locate and are not documented properly. | 2.5 Were efforts made to minimise errors in selection of studies? **Y**    Titles and abstracts were screened independently by two reviewers. Full text inclusion assessment was performed by two reviewers. Third reviewer was tiebreaker in the case of disagreements. | 3.5 Were efforts made to minimise error in risk of bias assessment? **N**  As mentioned above no ROB assessment tool was used. | 4.5 Were the findings robust e.g as demonstrated through funnel plot or sensitivity analyses? **Y**  Narrative synthesis- heterogeneity was addressed by not combining. |

Y= Yes, PY= probably yes, PN= probably no, N=no, NI= no information
