# Supplementary material for: The impact of leishmaniasis on mental health and psychosocial well-being: A systematic review
Source: PLoS One. 2019 Oct 17;14(10):e0223313. doi: 10.1371/journal.pone.0223313 (PMC6797112; doi:10.1371/journal.pone.0223313)
Supplement: S4 Table — (DOCX) [file pone.0223313.s007.docx]

**S4 Table- ROBIS Phase 3: Judging Risk of Bias**

| **Phase 3** | Concern |
| --- | --- |
| 1 Concerns regarding specification of study eligibility criteria | **Low-** No certainties of post-hoc changes to the eligibility criteria because as stated in 1.1 there was no published protocol. Restricting books and not including full-text could cause selection bias. Restricting by language could cause language bias. |
| 2. Concerns regarding methods used to identify and/or select studies | **Unclear-** Not enough databases were used. Regional databases could have been used to capture a wider scope of studies. Also, not including Portuguese as a language could bring concerns because many studies are likely to be published in Brazil. However, two separate reviewers independently with third reviewer as tiebreaker. |
| 3. Concerns regarding methods used to collect data and appraise studies. | **High-** No quality appraisal. |
| 4. Concerns regarding the synthesis and findings | **Low-** Despite lack of a protocol, the study discusses the results adequately describing different aspects of psychosocial burden found in the included studies. |
