# Supplementary material for: The impact of leishmaniasis on mental health and psychosocial well-being: A systematic review
Source: PLoS One. 2019 Oct 17;14(10):e0223313. doi: 10.1371/journal.pone.0223313 (PMC6797112; doi:10.1371/journal.pone.0223313)
Supplement: S5 Table — (DOCX) [file pone.0223313.s008.docx]

**ROBIS Phase 4**

| **Risk of Bias in the Review** |  |
| --- | --- |
| 1. Did the interpretation of findings address all of the concerns identified in Domains 1 to 4 | **PN** |
| 1. Was the relevance of the identified studies to the review’s research question appropriately considered? | **PY** |
| 1. Did the reviewers avoid emphasizing results on their basis of statistical significance | **PY** |

Y= yes, PY= probably yes, PN= probably no, N=no, NI= no information
